# Managing ulcerative colitis after surgery

**DOI:** 10.3389/fmed.2022.1081940

**Published:** 2023-01-04

**Authors:** Cristina Calvino-Suarez, Rocío Ferreiro-Iglesias, Iria Baston Rey, Manuel Barreiro-de Acosta

**Affiliations:** IBD Unit, Department of Gastroenterology, University Hospital Santiago de Compostela, Galicia, Spain

**Keywords:** pouchitis, ulcerative colitis, colectomy, antibiotics, biologic therapy

## Abstract

Management of ulcerative colitis after surgery suggested by guidelines (total proctocolectomy with ileal-pouch anal anastomosis) is a big challenge for physicians because patients who believed that their disease had been cured started experiencing very uncomfortable symptoms repeatedly. A high number of patients develop episodes of pouchitis, which is a non-specific inflammation of the pouch whose etiology is unknown. Antibiotics are the elective treatment for acute pouchitis, but regarding chronic pouchitis, this condition is very complicated to treat due to the absence of well-designed specific studies for this group of patients. Antibiotics, budesonide, and biological therapies are some of the recommended drugs for these patients, but despite their use, some need a permanent ileostomy.

## Introduction

Despite great advances in medical treatments, the risk of surgery in adults with ulcerative colitis (UC) is 4.4, 10.1, and 14.6% at 1, 5, and 10 years after diagnosis, respectively (1, 2). Total proctocolectomy with ileal pouch-anal anastomosis (IPAA) has become the surgical treatment of choice for a great number of patients. Surgical procedures help to control inflammatory activity, but there are associated risks and potential complications that must be taken into account when evaluating surgery.

Pouchitis is the most frequent non-specific inflammatory complication in the ileal pouch. The surgical procedure is divided into three sessions (two of which are performed in experienced centers). Despite the decrease in the number of cases of IPAA in patients with UC in the past years (3, 4), fundamentally related to the introduction of biological therapy, the risk of pouchitis in the first 2 years after IPAA has increased (5).

The main reason for the IPAA is to preserve continence and avoid permanent ostomies, but many complications can happen after the surgery. In patients with symptoms, pouch endoscopy is the best tool both for the diagnosis of pouchitis and for the differential diagnosis of other ileal pouch disorders, such as Crohn's disease of the pouch, infectious pouchitis, eosinophilic pouchitis, autoimmune pouchitis, irritable pouch syndrome, ischaemia, cuffitis, bacterial overgrowth, difficulty emptying the reservoir, afferent loop syndrome, and other malabsorption syndromes (6). Cuffitis is the inflammation of the rectal cuff in an operated UC. It is frequent in patients with IPAA and stapled anastomosis without mucosectomy, but it is also described in patients with manual suturing and mucosectomy (6). Table 1 shows the differential diagnosis of complications in patients with UC after total proctocolectomy with IPAA different from pouchitis and their treatment.

**Table 1:** Differential diagnosis complications in patients with UC after total proctocolectomy with IPAA different from pouchitis and their treatment.

**Complication**	**Main treatment**
Cuffitis	Topic Mesalamine
Irritable pouch syndrome	Behavioral intervention
Pouch ischemia	Hyperbaric oxygen therapy surgery
Pouch stenosis	Dilatation/revisional surgery
Pouch infection	Specific Infection treatment
Pelvic floor dysfunction	Ano-rectal physiology
Crohn's disease	Biologics

## Epidemiology and risk factors for pouchitis

Several complications can occur after total proctocolectomy with IPPA in patients with UC. Morbidities after IPAA are usually classified as early (within the first month after surgery) or late (occurring following the closure of ileostomy). The most frequent complication in long-term follow-up is pouchitis with a highly variable incidence of occurrence in different studies (7–59%) (5, 7). The cause of this great discordance could be the absence of universal diagnostic criteria and an etiology that is not yet fully known. The occurrence of this complication increases over the course of follow-up with cumulative incidence rates of 25, 36, and 45% at 1, 3, and 5 years, respectively (8, 9).

Some risk factors for developing pouchitis have been identified, such as the presence of extraintestinal manifestations [especially primary sclerosing cholangitis (PSC)], backwash ileitis, pancolitis, being a non-smoker, and the use of non-steroidal anti-inflammatory drugs (10–12). Among the most widely accepted etiopathogenic theories is the involvement of gut microbiota in the development of this complication. Many studies focusing on the pathogenesis of this morbidity found alterations in the microbiome such as dysbiosis with the altered balance of luminal bacteria or bacterial overgrowth in the pouch or even in the fecal microbial composition prior to colectomy. Moreover, the efficacy of antibiotics and probiotics in treating pouchitis supports the theory that the microbiome may play a role (13, 14).

## Diagnosis

The most common symptoms of pouchitis are watery diarrhea, abdominal pain, fever, tenesmus, fecal urgency, and incontinence, which can sometimes be associated with extra-intestinal manifestations (6). However, as this clinical setting is not specific to pouchitis, compatible endoscopic and histological findings are required to confirm the diagnosis and rule out other entities such as irritable pouch syndrome, concurrent surgery-related mechanical conditions, or cuffitis, for example (15).

Besides the careful evaluation of the pouch, the endoscopic examination should include the afferent ileal limb, the anastomosis, and the rectal cuff (if it exists). The main endoscopic features of pouchitis include erythema, friability, an absent vascular pattern, hemorrhage, oedema, erosions, and ulcerations (16). Along with the type of lesions, their distribution is also important since it can be useful in the differential diagnosis. Thus, the presence of inflammation limited to the distal half of the pouch suggests ischemia, whereas diffuse affection of the pouch together with a long segment of the afferent loop involvement is associated with autoimmune disorders and infections related to IgG4.

Some experts recommend taking biopsies from the pouch and the afferent limb regardless of whether macroscopic lesions are observed or not to detect mild endoscopic forms of pouchitis, cytomegalovirus (CMV) infection, Crohn's disease, ischaemia, and dysplasia. Nevertheless, taking biopsies from ulcers confined to the stapled line should be avoided since foreign-body granulomas can be misinterpreted as Crohn's disease of the pouch (17).

Histological findings of pouchitis usually include mucosal ulceration, cryptic abscesses, and neutrophilic infiltrates indicating acute inflammation, as well as chronic inflammation changes such as villous atrophy, crypt distortion, and chronic inflammatory infiltrates (18).

Although none of the existing indices for evaluating pouchitis have been fully validated, the most commonly used index is the Pouchitis Disease Activity Index (PDAI) (19). It is an 18-point score composed of symptom, endoscopy, and histology subscores, with each rated from 0 to 6. A total PDAI score of ≥ 7 points is considered a diagnosis of pouchitis.

## Natural history of pouchitis

Although the incidence of pouchitis has been shown to increase over time, in many cases, the first episode occurs within the first year after colectomy. The course of pouchitis resembles the course of UC and may appear with very sporadic flares or with periodic symptomatic exacerbations due to the presence of persistent inflammation in the pouch. Pouchitis is classified as acute when the duration of symptoms is <4 weeks and as chronic when the symptoms are longer than 4 weeks. It can also be classified as follows according to the number of exacerbations it presents: being infrequent (<3 episodes/year), recurrent (> 3 episodes/year), or continuous (20, 21).

The course of the disease is variable. It has been reported that up to 61% of patients will have a recurrence after the first episode of pouchitis, 39% will not relapse after one episode, and 5–19% will have refractory pouchitis (22, 23).

In most patients, medical treatment of pouchitis allows the persistence of the reservoir without the need for a long-term stoma. In a recent meta-analysis, the overall prevalence of pouch failure was 5%, further increasing to 9% in the group with more than 10 years of follow-up (24). Different risk factors are associated with reservoir failures, such as male gender, a high body mass index, advanced age, and the presence of extraintestinal manifestations (25).

Pouchitis can be complicated by the development of fistulae or abscesses, strictures, or malignancies. Cumulative incidences of pouch neoplasia were 1.3 and 5.1% at 10 and 25 years after colectomy, respectively. Therefore, endoscopic monitoring seems to be appropriate in these patients (26). However, this is a matter of controversy, with variations in follow-up recommendations between different societies. In 2017 ECCO guidelines, patients were classified as high risk if they had undergone colectomy due to cancer or dysplasia, had concomitant PSC, or had chronic pouchitis (27). In these high-risk patients, follow-up with annual endoscopy of the pouch was recommended, while in the remaining patients, there was insufficient evidence to recommend endoscopic surveillance. The Spanish group GETECCU, in their recommendations, suggested that those patients with a history of neoplasia or dysplasia before colectomy should be followed up with annual endoscopy, whereas those with chronic pouchitis, cuffitis, PSC, or family history of colon neoplasia should have an endoscopy every 1–3 years and those without risk factors, every 5 years (6). In consensus guidelines from the International Ileal Pouch Consortium recently published, pouch endoscopy is recommended every year in patients with dysplasia/cancer before surgery and endoscopy every 1–3 years for patients with any of the following risk factors: PSC, chronic pouchitis, or cuffitis; CD of the pouch, > 8 years from the diagnosis of UC or family history of colorectal cancer (first-degree relative). For patients without any risk factor, a surveillance interval has been suggested of no shorter than 3 years (28).

## Treatment

### Acute pouchitis

Depending on the duration of symptoms, acute pouchitis lasts <4 weeks. In 39% of patients, acute pouchitis never recurs (22).

The first-line of treatment is antibiotics, with response rates of near 80% (29). Clinical trials with a low number of patients support its use. The first clinical trial included 13 patients, although only 11 completed the crossover double-blind trial. The aim was to evaluate the efficacy of metronidazole vs. placebo for 2 weeks. Metronidazole demonstrated a statistically significant improvement in decreasing the number of bowel movements (30). It became the treatment of choice, but the multiple adverse effects led to the search for alternatives. Another small trial compared ciprofloxacin and metronidazole (31). For 2 weeks, 16 patients were randomized to receive 1,000 mg/day of ciprofloxacin vs. 20 mg/kg/day of metronidazole. Ciprofloxacin presented greater effectiveness with fewer adverse events. A few other antibiotics proposed were rifaximin, erythromycin, or amoxicillin-clavulanic acid, but with a low level of evidence. Therefore, ECCO guidelines (27) and GETECCU recommendations (6) suggest metronidazole or ciprofloxacin as the mainstay of treatment, although side effects are less frequent with ciprofloxacin.

Another treatment that has shown efficacy compared to metronidazole is budesonide enemas. In a prospective study of 26 patients, this topical steroid demonstrated similar clinical, endoscopic, and histological efficacy (32). In a non-controlled open-label trial of 23 patients, high doses of a specific probiotic mixture, called De Simone formulation, with eight bacterial strains that included Lactobacilli (*Lactobacillus casei, L. plantarum, L. acidophilus*, and *L. delbrueckii* ssp. *bulgaricus*), Bifidobacteria (*Bifidobacterium longum, B. breve*, and *B. infantis*), and *Streptococcus thermophilus* (currently called De Simone formulation), were effective in the treatment of mildly active pouchitis (33). In a non-controlled prospective study of 29 patients, 5-aminosalicylic acid (1.2–4 g per day) administered topically by suppositories or enemas demonstrated clinical and endoscopic improvement after 20–30 days of therapy (34). These options can be an alternative for patients with an intolerance to antibiotics.

### Prevention of chronic pouchitis

Patients with more than two annual episodes of pouchitis who have been treated with recommended antibiotic treatments can be considered antibiotic-dependent (6). The efficacy of the probiotic mixture (De Simone formulation) in this clinical context was shown in the study of Gionchetti et al. (35) (single dose of 6 g/day) and Mimura et al. (36) (dose of 3 g two times a day). Treatment with this probiotic formulation significantly reduced the risk of the reappearance of pouchitis in this type of patient. The small sample size and the lack of validation studies with this formulation make it difficult to establish a common recommendation for all patients. The use of other probiotics such as *L. rhamnosus GC*, despite demonstrating a change in the composition of the bacterial flora of the pouch, found no differences in clinical or endoscopic variables (37).

### Antibiotic chronic refractory pouchitis

An antibiotic combination is the elected choice for patients who develop chronic pouchitis.

In a systematic review, it was demonstrated that the strategy of a combination of antibiotics was effective with different types of antibiotics (38). A study with 16 patients combining both 1 g/day of ciprofloxacin and 15 mg/kg/day of tinidazole for 4 weeks showed an 88% remission rate compared with a 50% remission rate in a small group of 10 patients receiving mesalamine (39). In an open prospective study with 44 patients, the combination of metronidazole and ciprofloxacin showed a remission rate of more than 80% (40).

Locally active steroids have shown to be effective in these patients. Oral budesonide (9 mg/day for 8 weeks and reducing 3 mg/month) was administered to 20 antibiotic-refractory patients, of whom 3 out of 4 presented remission, which is defined according to a total PDAI score of ≤4 (41). Beclomethasone dipropionate (5 mg/day for 8 weeks) demonstrated remission rates of 80% in 10 patients using the same remission definition as in the previous study (PDAI score of ≤4) (42).

### Immunomodulators and biological therapy in chronic pouchitis

Despite their common use in clinical practice, most clinical guidelines cannot recommend the use of thiopurine immunomodulators in monotherapy for chronic pouchitis due to the absence of any kind of evidence (43).

The advent of anti-TNF drugs was a major breakthrough in the treatment of UC. Nevertheless, the introduction of these drugs in chronic pouchitis was slower than expected. Despite amazing remission rates (over 70%) with infliximab (IFX) (5 mg/kg at weeks 0, 2, 6, and every 8 weeks) in two independent series (44, 45), a multicentre study performed by the BIRD group, which included 28 patients with refractory pouchitis treated with IFX at a normal schedule, showed an 88% clinical response after induction but only 32% remission. Meanwhile, the PDAI decreased from 9 to 4.5 points (*p* <0.001). After a mean follow-up of 20 months, 56% of the patients presented a sustained clinical response, while 5 (17%) patients had to undergo permanent ileostomy (46). In the multicentre Spanish open-label study performed by Barreiro-de Acosta in 33 patients with chronic refractory pouchitis, only 21% of the patients achieved remission after induction, but 63% had a partial clinical response (47). Long-term remission rates after 1 year of treatment were 27%, with nearly 20% with a partial response. Adverse events were observed in 15% of patients, some of them probably due to immunogenicity because they had received IFX prior to surgery.

Regarding the efficacy of subcutaneous anti-TNF in chronic refractory pouchitis, data were scarce. A small open study with 8 refractory patients to IFX performed by GETECCU analyzed the efficacy of adalimumab, showing clinical response after induction in 38% of patiens and remission in only 13%. After 1 year, 50% of the patients avoided a permanent ileostomy, although only 25% achieved remission (48). A randomized, double-blind, placebo-controlled trial performed on 13 patients refractory to antibiotics showed no significant differences in the remission rates between adalimumab and placebo (49).

**Figure 1:**
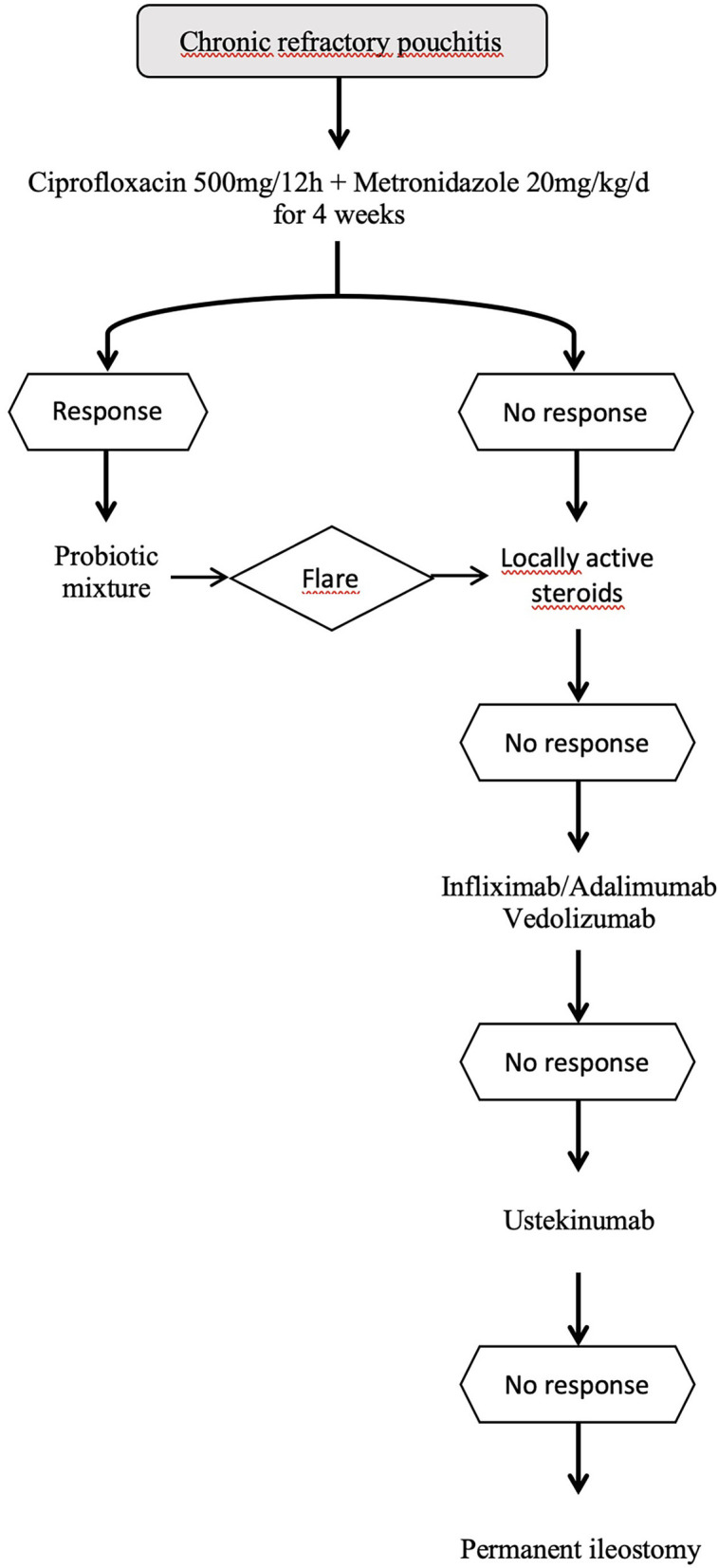
Algorithm for the treatment of chronic pouchitis.

Regarding other biological therapies such as vedolizumab, a couple of open-label multicentre studies with approximately 20 multi-refractory patients each that evaluated the efficacy of the drug in chronic refractory pouchitis showed a significant decrease in clinical symptoms measured by modified PDAI (50, 51). A meta-analysis evaluated all data with this drug and showed that clinical improvement at week 12 was obtained in 33 out of 44 patients (75%) (52). In a study from Leuven comparing infliximab, adalimumab, or vedolizumab, clinical remission was reported in 43.5, 38.5, and 60.0% of patients, respectively (53). A randomized clinical trial comparing vedolizumab with placebo was performed, and only abstract results were presented; in total, 102 patients were treated (51 per group), remission rates (comprising clinical symptoms and endoscopy domains) were 31.4% (*n* = 16/51) for vedolizumab vs. 9.8% (*n* = 5/51) for placebo at week 14 (54). The first data on the use of ustekinumab are from a single-center retrospective study with 24 patients that showed a 50% clinical response (55). Experience with other drugs such as tofacitinib is limited to clinical cases (56). Figure 1 shows an algorithm for the treatment of chronic refractory pouchitis.

### Surgical treatment

When medical treatment fails, pouch rescue surgery is an option. However, surgery in the treatment of pouchitis does not recommend a new pouch because of potential functional and post-surgery complications, with definitive ileostomy being the final solution in some of these refractory patients, with some psychological consequences for patients who have attempted to avoid in the past (57).

## Conclusion

The management of ulcerative colitis after surgery represents one of the biggest gaps in knowledge in inflammatory bowel disease treatment, especially if the patient develops pouchitis. A clear differential diagnosis is one of the keys to the management of these complications. Due to the lack of randomized studies and the scarce number of patients per center, the management of these patients is difficult, and it is therefore of the utmost importance to report experiences with new drugs to help colleagues in the future. If a patient does not achieve remission with one drug, we recommend changing quickly to the next option according to the proposed algorithm to avoid facing irreversible situations.

## Author contributions

CC-S, RF-I, and IB have wrote different parts. MB-d concieved and reviewed the final version. All authors contributed to the article and approved the submitted version.
